# Fate of human mesenchymal stem cells (MSCs) in humans and rodents—Is the current paradigm obtained on rodents applicable to humans?

**DOI:** 10.1111/jcmm.13561

**Published:** 2018-02-14

**Authors:** Jan Lakota

**Affiliations:** ^1^ Biomedical Center, SAS Bratislava Slovakia; ^2^ Institute of Normal and Pathological Physiology CEM, SAS Bratislava Slovakia; ^3^ St. Elizabeth Cancer Institute Bratislava Slovakia

Dear Editor,

Mesenchymal stem cells (MSCs) are multipotent stromal cells that can differentiate into a variety of cell types (osteoblasts, chondrocytes, myocytes and adipocytes). MSCs are found in bone marrow, adipose fat tissue, umbilical cord, dental pulp and other tissues [for review, see^1^]. They are able to regenerate different damaged tissues.^1^ There is an intensive research of the use of MSCs in the tissue regeneration and the disease treatment, which is performed on animals, mainly rodents. Moreover, it has been shown that in rodents (mice, rats), the MSCs migrate directly to the damaged, inflamed areas, including tumours.^2^, ^3^ This phenomenon has been used to prepare “therapeutic” MSCs. The “therapeutic” MSCs are genetically modified MSCs that contain stable gene coding for protein or enzyme product able to kill the tumour cells. An example of such construct is the yeast enzyme cytosine deaminase, which converts the rather non‐toxic 5‐fluorocytosine to the cytostatic agent 5‐fluorouracil. In the presence of 5‐fluorocytosine, the MSCs, which (in rodents) invade in the tumour, were able to kill the tumour cells and to “cure” the animals.^4^ This therapeutic concept became known as the “stem cell‐based cancer gene therapy”.^5^ The main “ideological” obstacle of the MSC use (normal or genetically modified) is the observation that the intravenous infusion of MSCs generally leads to their entrapment in the lung, liver and spleen.^6^, ^7^ However, this experimental fact is based on the data obtained on rodents (mice, rats). We used the “therapeutic” MSCs to treat a patient with squamous carcinoma of the tongue who developed lung metastases. There was no sign of any therapeutic effect after intravenous (not local, ie intratumour) administration of the “therapeutic” MSCs. (The CT scan performed on day +6 (the “therapeutic” MSCs were applied on the day 0) showed no difference in the size or density of the patients' pulmonary metastases compared to the CT scan on day −1. On the day +40, there were signs of a progression of the metastases on the CT scan.) After the intravenous administration, the “therapeutic” MSCs were “homing” in the bone marrow. Even a rather low cell count was able to cause grade 2 thrombocytopenia and grade 3 neutropenia, respectively.^8^ We conclude that there has not been any entrapment of the “therapeutic” MSCs in the lungs. Nor the cells were homing in tumour metastases. However, even a small number (60 × 10^6^ cells) of the “therapeutic” MSCs were able to cause a deep temporal bone marrow suppression. We have performed a treatment of another patient with metastatic (liver, retroperitoneum, abdominal wall) ovarian carcinoma. The “therapeutic” MSCs were applied locally into two metastases in the abdominal wall. We have observed only partial necrosis of these two metastases. The other metastases progressed without any sign of temporal regression, and the patient finally died of liver failure. There was no sign of any therapeutic effect of the “therapeutic” MSCs on other patients' metastases. We concluded that the “therapeutic” MSCs did not migrate to any “neighbour” metastases (liver, retroperitoneum, abdominal wall) (Lakota J, unpublished observation). The indirect confirmation that the MSCs (or “therapeutic” MSC) are in humans not entrapped in the lung, liver and spleen comes from thousands haematopoietic stem cell transplantations performed annually in patients with malignant disease. A considerable part of these transplants is performed with the stem cells obtained from bone marrow. The bone marrow is rich in haematopoietic as well as mesenchymal stem cells. So far, nobody observed any entrapment of these cells in the patient's lungs. In our opinion, the requisitioned data of the MSCs, mechanically taken from mice and rats, could negatively influence the trends of the research in the novel treatment(s) of human diseases.

## CONFLICT OF INTEREST

The author confirms that there is no conflict of interests.
